# Hydrocarbon-Based Composite Membrane Using LCP-Nonwoven Fabrics for Durable Proton Exchange Membrane Water Electrolysis

**DOI:** 10.3390/polym15092109

**Published:** 2023-04-28

**Authors:** Seok Hyeon Kang, Hwan Yeop Jeong, Sang Jun Yoon, Soonyong So, Jaewon Choi, Tae-Ho Kim, Duk Man Yu

**Affiliations:** 1 Energy Materials Research Center, Korea Research Institute of Chemical Technology (KRICT), Daejeon 34114, Republic of Korea; mlc1207@krict.re.kr (S.H.K.); hwanyeop@krict.re.kr (H.Y.J.); sjyoon@krict.re.kr (S.J.Y.); syso@krict.re.kr (S.S.); 2 Department of Polymer Science and Engineering, Kyungpook National University, Daegu 41566, Republic of Korea

**Keywords:** water electrolysis, proton exchange membrane, composite membrane, hydrocarbon-based ionomer, LCP-nonwoven fabric

## Abstract

A new hydrocarbon-based (HC) composite membrane was developed using liquid crystal polymer (LCP)-nonwoven fabrics for application in proton exchange membrane water electrolysis (PEMWE). A copolymer of sulfonated poly(arylene ether sulfone) with a sulfonation degree of 50 mol% (SPAES50) was utilized as an ionomer for the HC membranes and impregnated into the LCP-nonwoven fabrics without any surface treatment of the LCP. The physical interlocking structure between the SPAES50 and LCP-nonwoven fabrics was investigated, validating the outstanding mechanical properties and dimensional stability of the composite membrane in comparison to the pristine membrane. In addition, the through-plane proton conductivity of the composite membrane at 80 °C was only 15% lower than that of the pristine membrane because of the defect-free impregnation state, minimizing the decrease in the proton conductivity caused by the non-proton conductive LCP. During the electrochemical evaluation, the superior cell performance of the composite membrane was evident, with a current density of 5.41 A/cm^2^ at 1.9 V, compared to 4.65 A/cm^2^ for the pristine membrane, which can be attributed to the smaller membrane resistance of the composite membrane. From the results of the degradation rates, the prepared composite membrane also showed enhanced cell efficiency and durability during the PEMWE operations.

## 1. Introduction

Hydrogen generation systems using natural gas reforming, autothermal reforming, and water electrolysis have been significantly improved for fuel cells to generate electricity, power, and heat [1,2]. Among these hydrogen production methods, proton exchange membrane water electrolysis (PEMWE), in which hydrogen and oxygen gases are obtained at the cathode and anode, respectively, has the advantage of being a simple and environmentally friendly process that produces high-purity hydrogen at a relatively low operating temperature [3]. The proton exchange membrane (PEM) plays a crucial role as a component in PEMWE because it acts as an electrical insulator that prevents electrons from passing across the membrane, a separator that prevents gas crossing, and a medium that selectively transmits protons [4,5,6].

Currently, perfluorinated sulfonic acid (PFSA) ionomers, such as Nafion, are widely employed as the predominant PEM materials because of their high proton conduction and chemical stability [7,8]. However, this material has considerable limitations, such as the high production cost and limited operating temperature, making the commercial use of this membrane difficult for high-performance PEMWE [9]. Therefore, numerous studies have been conducted on inexpensive hydrocarbon-based (HC) ionomers capable of overcoming the drawbacks of PFSA ionomers [10,11,12,13,14]. In particular, sulfonated poly(arylene ether sulfone) (SPAES) copolymers have been extensively studied as HC ionomers due to their outstanding characteristics, such as high proton conductivity and remarkable chemical and thermal stability [15,16,17,18]. For example, Park et al. [19] synthesized an SPAES copolymer with a sulfonation degree of 50 mol% (SPAES50) for use as a PEM in PEMWE. The thin (20 μm) SPAES50 membrane-based PEMWE exhibited outstanding performance (1.07 A/cm^2^ at 1.6 V), superior to that of the Nafion-based PEMWE. This result arose from the lower ohmic resistance of the SPAES50 membrane owing to its smaller membrane thickness. Han et al. [20] also reported random and multi-block types of SPAES copolymers and analyzed their performances and degradation rates (DRs) in PEMWE. At a similar ion exchange capacity (IEC), the selectivity of protons to hydrogen for the random SPAES membrane was higher than that for the multiblock SPAES membrane because of the less-separated hydrophilic channels. For the single-cell test, better performance was observed in the random SPAES membrane at 1.9 V (5.3 A/cm^2^) compared with that in the Nafion 212 membrane (4.8 A/cm^2^). However, the DR of the SPAES membrane (951 μV/h) was found to be higher when compared to the Nafion membrane (613 μV/h) at alternating current densities of 3 and 0.02 A/cm^2^. Consequently, it was demonstrated that the SPAES membranes have the potential for use in PEMWE as alternatives to the Nafion membrane. However, the lower durability of these SPAES membranes at high IECs must be addressed and overcome for long-term PEMWE operation [21,22,23].

An effective approach to improve the durability of SPAES membranes is the use of porous substrates that are insoluble in aprotic polar solvents, such as *N*-methyl-2-pyrrolidone (NMP) and *N*,*N*-dimethylacetamide (DMAc). As the dimensional variation of an SPAES membrane increases significantly in water at a high IEC over 1.5 meq/g, where the SPAES membrane exhibits proton conductivity similar to or better than that of the Nafion membrane, porous substrates are essential to reduce water swelling and improve the mechanical strength of the SPAES membrane for a highly robust PEM [17,24,25,26,27]. In addition, the membrane thickness can be reduced using porous substrates, lowering the membrane resistance and the amount of the ionomer to achieve excellent cell performance and low costs [28,29]. Recently, Hong et al. [30,31] designed an SPAES-based composite membrane with a chemically and mechanically stable poly(tetrafluoroethylene) (PTFE) substrate. As porous PTFE possesses a low surface energy, which typically hinders the impregnation of the HC ionomer dissolved in NMP or DMAc, the surface of the PTFE was modified using an n-propyl alcohol (NPA) solvent. The NPA solvent effectively mediated the interfacial interactions between the SPAES solution and PTFE and enabled the penetration of the SPAES ionomer into the pores of the PTFE. As a result, the SPAES composite membranes showed outstanding dimensional stability and mechanical properties owing to the reinforcing effect of PTFE. During the PEMWE operation, lower gas permeability and DR were also observed at a constant current density of 2 A/cm^2^ in the composite membranes than in the pristine membranes, indicating that the porous PTFE successfully suppressed the swelling of the SPAES ionomers at 80 °C in water. Noh et al. [32] developed composite membranes composed of SPAES and PTFE with three and five layers, using PTFE substrates of 10 and 5 μm thickness, respectively, to investigate the impact of PTFE thickness on the impregnation state of the SPAES ionomer. When the PTFE substrate with a thickness of 5 μm was used for the composite membrane, noticeable defects were not observed in the composite layer, indicating that a thinner PTFE is better for the impregnation of the SPAES ionomer. As a result of the robust interlocking between the two components, the five-layered composite membrane exhibited greater dimensional stability and mechanical toughness when compared to the three-layered composite membrane. For the electrochemical properties in the PEMWE operation, the highest current density was obtained from the five-layered composite membrane, which showed a 1.6-fold improvement at 1.9 V compared to the pristine membrane, owing to the smaller membrane resistance. Moreover, the DR of the five-layered composite membrane was 330 μV/h, which was a quarter of that of the pristine membrane. Consequently, the use of porous substrates for SPAES membranes was determined to be an effective way to enhance the cell performance and durability of PEMWE.

In this study, a new composite membrane was fabricated by incorporating liquid crystal polymer (LCP)-nonwoven fabrics into an SPAES ionomer. As the LCP consists of rigid aromatic polymer chains, the LCP nonwoven material has outstanding mechanical properties [33]. In addition, it was not dissolved in NMP or DMAc, meaning that the impregnated SPAES solution did not deform the pores of the LCP-nonwoven fabrics. In contrast to the SPAES/PTFE composite, the SPAES/LCP composite was prepared via a simple impregnation process without any surface treatments of the LCP-nonwoven material owing to the small difference between the surface energies of the SPAES solution and LCP because of their hydrocarbon-based structures. For the SPAES ionomer, the degree of sulfonation was adjusted to 50 mol% and the IEC value was found to be 1.87 meq/g. To compare the SPAES50 membrane with the composite membrane, the water uptake, dimensional variation, mechanical properties, and proton conductivity were examined at different temperatures under wet conditions. Subsequently, the electrochemical properties, such as the single-cell performance, resistance, and DR, were evaluated at 80 °C with a water flow rate of 30 mL/min and compared to those of the Nafion 212 membrane.

## 2. Materials and Methods

### 2.1. Materials

4,4′-difluorodiphenylsulfone (DFDPS) and 4,4′-dihydroxybiphenyl (BP) were purchased from Richem International Ltd., Weifang, China, and Songwon Industrial Co., Ltd., Ulsan, Republic of Korea, respectively. Prior to their utilization, both the DFDPS and BP were subject to recrystallization from ethanol. The synthesis of 3,3′-disulfonated-4,4′-difluorodiphenyl sulfone (SDFDPS) was carried out using a procedure previously reported in the literature, which involved the use of fuming sulfuric acid (65% concentration; Merck, Darmstadt, Germany) [34,35]. NMP, toluene, and potassium carbonate (K_2_CO_3_) were purchased from Sigma-Aldrich (St. Louis, MO, USA), and the K_2_CO_3_ was dried at 80 °C for 24 h under vacuum before use. Porous LCP-nonwoven fabrics (porosity > 60%, thickness = 20 ± 2 µm) were obtained from Kuraray, Tokyo, Japan. Iridium oxide (IrO_2_) and Pt/C powders were purchased from Boyaz Energy, Seoul, Republic of Korea, and Tanaka Kikinzoku, Tokyo, Japan, respectively.

### 2.2. Synthesis of the SPAES50 Copolymer

**Figure 1:**
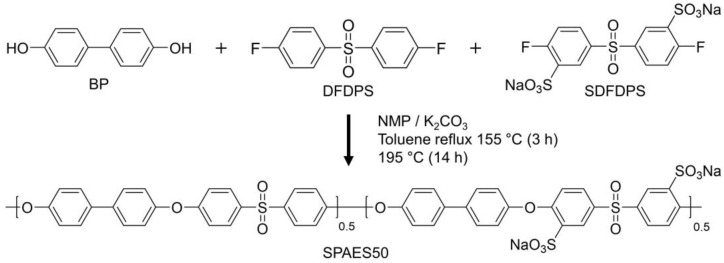
Synthetic scheme of the sulfonated poly(arylene ether sulfone) copolymer with a degree of sulfonation of 50 mol% (SPAES50).

SPAES50 (with a degree of sulfonation of 50 mol%) was synthesized via a condensation polymerization process involving BP and equimolar amounts of DFDPS and SDFDPS, as illustrated in Figure 1 [36,37]. First, the BP (6.8649 g, 0.0369 mol) and K_2_CO_3_ (6.1653 g, 0.0446 mol) were dissolved in NMP (40 mL). This solution was placed into a 250 mL four-neck flask reactor equipped with a mechanical stirrer, condenser, dean-stock trap, and a nitrogen inlet, and toluene (40 mL) was added as an azeotropic agent. The mixture was slowly heated to 155 °C for 2 h, and the temperature was maintained for 4 h to completely dehydrate the reaction system. The temperature was gradually increased to 185 °C and maintained at this temperature for 2 h to remove the toluene. After this, the mixture was cooled down to 167 °C, and then DFDPS (4.6866 g, 0.0184 mol) and SDFDPS (8.4486 g, 0.0184 mol) were added to the reactor, along with 40 mL of NMP. When the reactor temperature reached 195 °C for polymerization, a viscous solution was obtained after 13 h at the same temperature. Subsequently, the reactor was cooled to room temperature and diluted with NMP for filtration. The resulting polymer was thoroughly washed multiple times with isopropyl alcohol and deionized (DI) water to remove any remaining salt and unreacted monomers. Subsequently, SPAES50 powder was obtained by drying it in a vacuum oven at 80 °C for 24 h.

### 2.3. Preparation of the SPAES/LCP Composite Membrane

**Figure 2:**
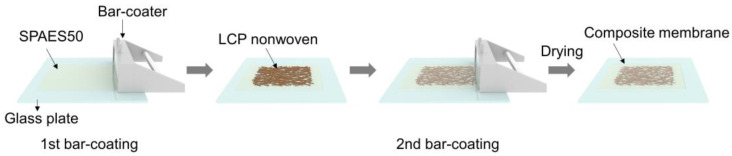
Schematic diagram of the preparation of the SPAES50/liquid crystal polymer (LCP) nonwoven composite membranes using a bar-coating method.

The SPAES50 solution, dissolved in NMP (20 wt%), was applied to a clean glass substrate using a doctor blade to ensure the uniformity of the membrane thickness. The porous LCP-nonwoven material was then placed onto the cast film, as illustrated in Figure 2. Next, the SPAES50 solution was cast onto the LCP-nonwoven material again and dried in an oven at 80 °C for 4 h. To convert the salt form of the sulfonate groups into the protonated form, the prepared composite membrane was immersed in 1.5 M sulfuric acid at 30 °C for 24 h, and subsequently, washed with DI water at room temperature for 24 h. The protonated composite membrane was obtained after drying on a vacuum plate for 6 h (thickness = 33 ± 2 µm) and denoted as L-SP50.

### 2.4. Characterization of the Membranes

The cross-sectional morphology of the composite membrane was examined using a scanning electron microscope (SEM; Vega II LSU, TESCAN, Brno, Czech Republic) after sputter coating with platinum (SC7640, Quorum Technologies, Lewes, United Kingdom) under vacuum for 2 min prior to observation. To examine the pore size distribution of the LCP-nonwoven fabrics, an advanced capillary flow porometer (CFP-1500AEL, wet up/dry up mode, Porous Materials, Ithaca, NY, USA) was used with the Galwick solution. The water uptake capacity and dimensional variation of the membranes were obtained from the weight and volume changes in the specimens (2 cm square, four specimens for each membrane) before and after immersion in DI water at 25, 50, and 80 °C. The mechanical properties of the membranes were measured using a material testing machine (LR5K, Lloyd Instruments, Bognor Regis, UK) with a crosshead speed of 5 mm/min at room temperature under hydrated conditions. Five dog bone-shaped specimens were tested, and the average values of their tensile strength and Young’s modulus were calculated. The IEC was determined using an automatic titrator (888 Titrando, Metrohm, Herisau, Switzerland). The membrane samples were immersed in 1 M NaCl solution at room temperature for 24 h to substitute the protons of the sulfonic acid groups with sodium ions. Then, the released protons in the solution were titrated with a 0.01 M NaOH solution using a pH meter. The in-plane proton conductivity of the membranes was evaluated using a four-probe conductivity cell, with an AC amplitude of 14.2 mV and a frequency range of 100 mHz to 200 kHz. The through-plane proton conductivity was measured using a two-plate conductivity cell, with an AC amplitude of 420 mV and a frequency range of 10 mHz to 1 MHz. All the measurements were taken after establishing the equilibration of the membranes in DI water for 6 h, and the proton conductivity (σ) was calculated as follows:

(1) $$\sigma (S/cm)=\frac{l}{R\times S}$$

where *l* is the distance between the two electrodes, and *R* and *S* are the resistance and cross-sectional surface area values of the membrane, respectively [38,39].

### 2.5. Preparation of the Membrane Electrode Assemblies (MEAs)

IrO_2_ powder, Nafion ionomer solution, NPA, and DI water were employed as catalyst slurries for the anode. The ratio of IrO_2_ to Nafion was adjusted to 9:1, and the solid content was 40 wt%. The cathode catalyst slurry was fabricated using Pt/C (46.6 wt% Pt) powder, Nafion ionomer solution, DI water, and NPA. The ratio of Pt/C to Nafion ionomer was adjusted to 75:25, and the solid content was 17 wt%. For the catalyst layer (CL), the catalyst slurry was sonicated for 10 min and bar-coated onto a fluorinated polyimide (FPI) film. Afterwards, the prepared CL was dried in an oven at 70 °C for 2 h and cut into squares of 5 cm^2^. To fabricate MEA, the membrane was sandwiched between the CLs and hot-pressed for 10 min at 130 °C and 120 kgf/cm^2^. The FPI films were then removed from the CLs (called the decal transfer method) [32]. The IrO_2_ and Pt loadings were 2.4 ± 0.2 and 0.36 ± 0.02 mg/cm^2^, respectively.

### 2.6. Electrochemical Measurements of PEMWE

To determine the PEMWE performance, the single-cell was assembled with MEA placed in the center, a Pt-coated titanium transport layer (Pt-coated Ti-PTL; Bekaert, Zwevegem, Belgium) on the anode, and a gas diffusion layer (GDL; Sigracet 39 BC, SGL carbon, Wiesbaden, Germany) on the cathode. Electrochemical measurements were performed using a PEMWE station (PWETS-001, CNL Energy, Seoul, Republic of Korea) with a water flow rate of 30 mL/min at 80 °C. To activate the cell, a voltage of 1.55 V was applied for 30 min, and then the polarization curves were obtained between 1.35 and 2.0 V with a step of 50 mV per 30 s. Electrochemical impedance spectroscopy (EIS) measurements (HCP-803, Biologic, Orlando, FL, USA) were taken at a DC potential of 1.7 V in the frequency range of 50 mHz to 30 kHz with an amplitude of 5 mV. For the durability test, a constant current density of 2 A/cm^2^ was applied to the cell for 100 h, and the increased voltage rate was recorded as a DR.

## 3. Results and Discussion

### 3.1. Morphology

**Figure 3:**
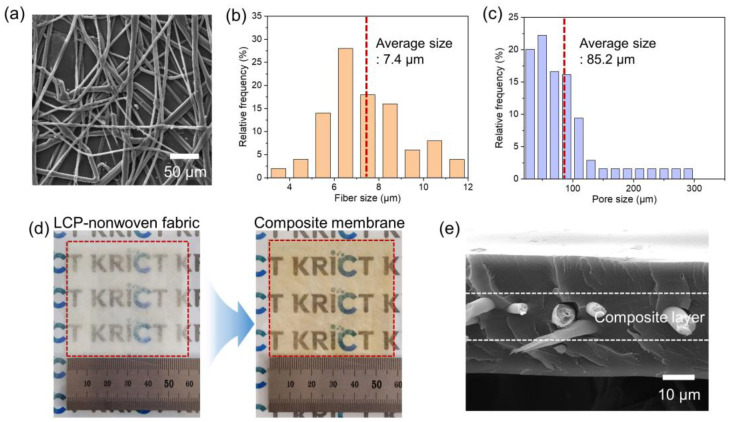
(**a**) Surface SEM image and (**b**) fiber and (**c**) pore size distributions of the LCP-nonwoven material. (**d**) Digital photographs of the surfaces of the LCP-nonwoven material and SPAES50/LCP composite membrane. (**e**) Cross-sectional SEM image of the SPAES50/LCP composite membrane.

SPAES membranes with a high IEC of 1.5 meq/g have been reported as high-performance PEMs, although they are vulnerable to dimensional variations in water, resulting in poor physical durability. In this study, the SPAES50 copolymer was synthesized via condensation polymerization, and the chemical structure and sulfonation degree were verified by means of ^1^H NMR spectroscopy in DMSO-d_6_, as shown in Figure S1. The actual sulfonation degree of the SPAES50 was calculated to be 46.6% from the integral ratio of the proton peaks, and its IEC was found to be 1.87 meq/g. To enhance the dimensional and mechanical stability, porous LCP-nonwoven material was introduced into the SPAES50 membrane using a simple impregnation process. Figure 3a,b show the surface morphology of the LCP-nonwoven material and the fiber size distribution, respectively. The average fiber size was 7.4 μm, and randomly aligned fibers without any significant defects were observed. In addition, the average pore size was approximately 85.2 μm, indicating micro-scaled pores (Figure 3c). The porosity of the LCP-nonwoven material was characterized using the 1-butanol uptake method [40], and it was calculated to be 68%. Figure 3d shows digital photographs of the surfaces of the LCP-nonwoven material and composite membrane (6 cm square). The opaque surface of the LCP-nonwoven material was changed to a transparent surface after the SPAES50 penetration into the porous LCP-nonwoven material owing to a decrease in the difference in the refractive index between the LCP and SPAES50 in the pores of the LCP. This demonstrates that the SPAES50 was completely impregnated into the LCP-nonwoven material without any surface treatment, and this facile process was effective in generating a composite membrane using an HC ionomer. The cross-sectional morphology of the composite membrane was also observed, as shown in Figure 3e. The SPAES50/LCP composite layer was located in the middle of the SPAES50 layers, and noticeable defects were not observed in the composite layer, indicating that a three-layered structure was successfully developed using the LCP-nonwoven material and SPAES50 ionomer.

### 3.2. Water Uptake and Dimensional Change

**Figure 4:**
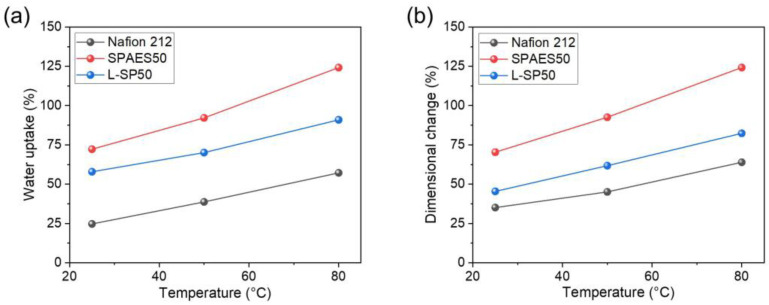
(**a**) Water uptake and (**b**) volumetric dimensional changes of the Nafion 212, SPAES50, and L-SP50 membranes at 25, 50, and 80 °C in water.

The water uptake capacity and swelling behavior are crucial to the proton-conducting properties and physical robustness, respectively, of a membrane used in PEMWE. The proton conductivity of a membrane is improved by the absorption of a large amount of water, as the hydrated ionic clusters within the membrane facilitate the transfer of protons through the diffusion of protonated water molecules. However, excessive water uptake leads to a decrease in dimensional stability, resulting in poor durability caused by mechanical failure or delamination between the membrane and the CL [41,42]. Therefore, it is necessary to minimize the dimensional change in the membrane to enhance its durability. Figure 4 shows the water uptake and volumetric dimensional variations of the Nafion 212, SPAES50, and L-SP50 membranes at 25 °C, 50 °C, and 80 °C in water. With increasing temperature, the water uptake capacities of all the prepared membranes increased, and the SPAES50 membrane exhibited the highest value at all the temperatures. The water uptake capacities of the Nafion 212 membrane were 25 and 57% at 25 and 80 °C, respectively, which were less than half of those of the SPAES50 membrane (72 and 124% at 25 and 80 °C), arising from the lower IEC of the Nafion 212 (0.90 meq/g) when compared with the SPAES50. As expected, the volumetric dimensional changes in the membranes showed trends similar to those of the water uptake because the membrane swelling depended on the water content in the membrane. The dimensional changes in the Nafion 212 and SPAES50 membranes at 80 °C were 64 and 124%, respectively. For the L-SP50 membrane, the water uptake and dimensional change at 80 °C were 91 and 82%, respectively, which were smaller than those of the SPAES50 membrane. This is because the LCP-nonwoven material was not swollen in water and the ionomer swelling was simultaneously suppressed by the LCP fabrics. In particular, a larger change was observed in the length compared with that in the thickness, owing to the pore direction of the LCP-nonwoven fabrics, as shown in Figure S2. The change in the length of the L-SP50 membrane was 18%, which was similar to that of the Nafion 212 membrane (16%). From these results, it was demonstrated that the interlocking structure between the SPAES50 ionomer and LCP-nonwoven fabrics was well developed, enabling effective suppression of membrane swelling.

### 3.3. Mechanical Properties

**Figure 5:**
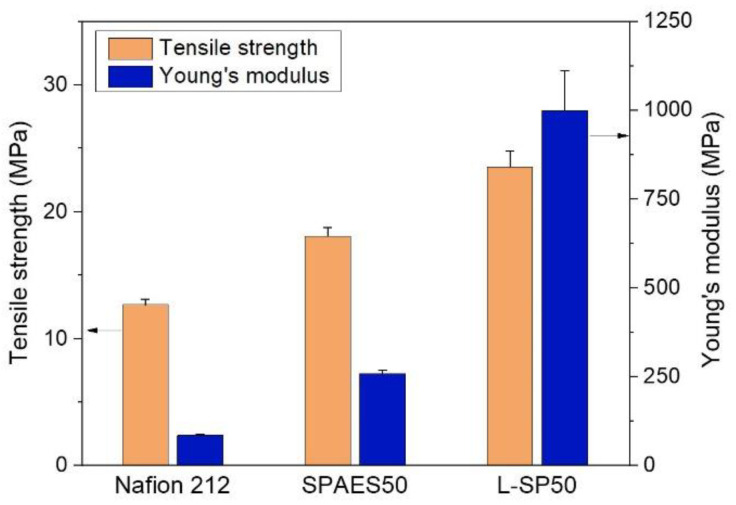
Tensile strengths and Young’s moduli of the Nafion 212, SPAES50, and L-SP50 membranes at room temperature under wet conditions.

A PEM must be mechanically robust to ensure long-term PEMWE operations. As PEMWE operates under fully hydrated conditions, mechanical tests of the membranes were performed with the specimens in a wet state. Figure 5 shows the tensile strength and Young’s moduli of the Nafion 212, SPAES50, and L-SP50 membranes at room temperature. The SPAES50 membrane exhibited a tensile strength of 18.1 MPa and a Young’s modulus of 258.0 MPa, which were 47% and 202% higher than those of the Nafion 212 (12.7 and 85.5 MPa), respectively, owing to the rigid backbone structure. As the LCP-nonwoven material was introduced into the SPAES50, the mechanical properties of the L-SP50 membrane improved by suppressing the membrane swelling, and the highest tensile strength and Young’s modulus (23.5 and 999.7 MPa) were obtained. Notably, a drastic increase in the Young’s modulus was observed when compared to the pristine membranes, inhibiting membrane deformation when it encountered an accidental force. Thus, the composite membrane achieved outstanding mechanical properties because the SPAES50 ionomer was well interlocked with the LCP-nonwoven material, as observed in the cross-sectional morphology.

### 3.4. Proton Conductivity

**Figure 6:**
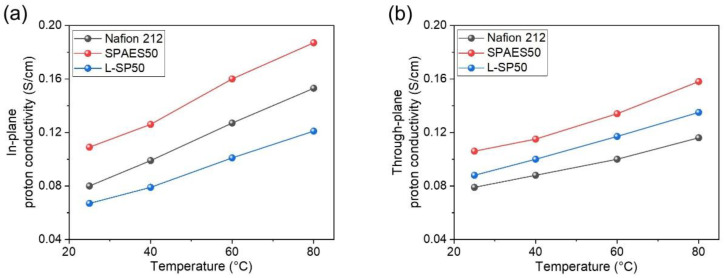
Proton conductivities of the Nafion 212, SPAES50, and L-SP50 membranes in (**a**) the in-plane and (**b**) the through-plane directions at 25–80 °C in water.

In PEMWE, protons are transported across the membrane in a direction from the anode to the cathode, and hydrogen is produced from a pair of protons in the cathode. Therefore, the proton conductivity of the membrane plays a crucial role in determining the efficiency and performance of PEMWE. Figure 6 shows the proton conductivities of the Nafion 212, SPAES50, and L-SP50 membranes as evaluated in two directions and at temperatures ranging from 25 to 80 °C in water. The proton conductivities of all the membranes showed an increase with a rising temperature. In the in-plane direction, the proton conductivities of the SPAES50 membrane were 0.109 and 0.187 S/cm at 25 and 80 °C, respectively, which were the highest values among the membranes. However, the L-SP50 membrane exhibited a notable reduction in proton conductivity because the LCP-nonwoven material was not proton-conducting. The in-plane proton conductivity of the L-SP50 membrane was 0.121 S/cm at 80 °C, which was lower than that of the Nafion 212 (0.153 S/cm). In contrast, the L-SP50 membrane exhibited higher proton conductivity compared to the Nafion 212 membrane in the through-plane direction. This is because the pores of the porous substrates are normally developed in the through-plane direction, indicating the easier passage of protons in the through-plane direction compared to the in-plane direction of the composite membrane. In addition, when the membrane was applied to a single-cell, the movement of protons was observed to be predominantly in the through-plane direction, confirming that the through-plane proton conductivity directly influenced the cell performance of the composite membrane with the porous substrate. The through-plane proton conductivity of the L-SP50 membrane was found to be 0.135 S/cm at 80 °C, which was 15% lower than that of the SPAES50 membrane. This difference between the two membranes was smaller than that seen in relation to the in-plane conductivity (35%) owing to the pore direction of the LCP-nonwoven fabric. From these results, the through-plane measurement was confirmed to be a more accurate method for evaluating the proton conductivity of the composite membrane than the in-plane measurement [43].

### 3.5. Electrochemical Performance of PEMWE

**Figure 7:**
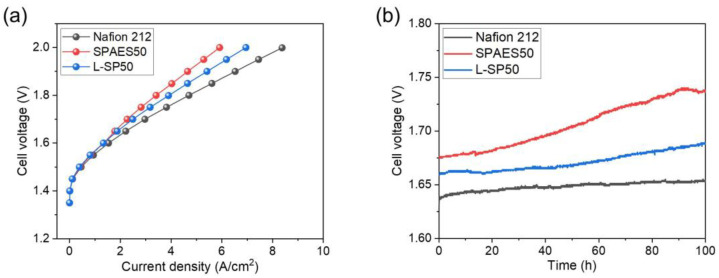
(**a**) PEMWE single-cell performances of the Nafion 212, SPAES50, and L-SP50 membranes (operating temperature: 80 °C; potential range: 1.25−2.0 V; active area: 5 cm^2^). (**b**) Voltage changes in the prepared membranes when a constant current density of 2 A/cm^2^ was applied to the cell for 100 h.

For assessing the electrochemical performance of PEMWE, the MEA was prepared using a decal transfer method, and it was assembled with diffusion layers, rubber gaskets, and bipolar plates as a single-cell. The current–voltage polarization curves of the single-cells were measured by varying the voltage from 1.35 to 2.0 V with a water flow rate of 30 mL/min at 80 °C. Figure 7a shows the PEMWE performances of the Nafion 212, SPAES50, and L-SP50 membranes. Even though the SPAES50 membrane had higher proton conductivity, its current density of 4.65 A/cm^2^ at 1.9 V was lower than that of the Nafion 212 membrane (6.53 A/cm^2^ at 1.9 V). This may arise from the large dimensional variation in the SPAES50 membrane in the thickness direction, which increases the membrane resistance. For the L-SP50 membrane, the current density at 1.9 V (5.41 A/cm^2^) was higher than that of the SPAES50 membrane despite of its lower proton conductivity, because the L-SP50 membrane (33 ± 2 µm) was thinner than the SPAES50 membrane (47 ± 2 µm), resulting in lowered membrane resistance. Moreover, the L-SP50 membrane showed higher current density than the recently studied HC membranes [10,19,44,45]. Thus, a composite membrane with a porous substrate has the advantage of possessing reduced membrane thickness, as mentioned above. However, the L-SP50 membrane exhibited inferior cell performance when compared to the Nafion 212 membrane because the LCP-nonwoven material was not effective in preventing membrane swelling in the thickness direction. Additionally, EIS measurements were conducted to examine the resistances of the membranes, as shown in Figure S3. In the EIS curve, the x-intercept on the left side of the semicircle in the high-frequency region and the diameter of the semicircle provide information about the ohmic resistance (*R_Ω_*) and charge transfer resistance (*R_ct_*), respectively. The *R_Ω_* is primarily influenced by the resistances associated with proton and electron transport and the interfacial contact between different components in the cell. On the other hand, the *R_ct_* is primarily attributed to the oxygen evolution reaction that occurs at the anode [46,47]. At a voltage of 1.7 V, the overall resistance of the L-SP50 membrane was 0.076 Ω cm^2^, which was lower than that of the SPAES50 membrane (0.095 Ω cm^2^) owing to its smaller thickness. The Nafion 212 membrane exhibited the lowest overall resistance (0.063 Ω cm^2^), correlating with the single-cell performance results. For the durability tests, the voltage of the MEA was continuously monitored for 100 h while applying a current density of 2 A/cm^2^. Figure 7b shows the voltage changes in the Nafion 212, SPAES50, and L-SP50 membranes and their increased voltage rates as the DRs for 100 h were calculated. A gradual increase in voltage for all the membranes was observed with an increasing time, indicating a decrease in the efficiency of the single-cell. In addition, no mechanical failure of the membranes was observed, which would be identified from a sharp drop in voltage [32]. The DR of the SPAES50 membrane was found to be 620 μV/h, which was roughly three-fold higher than that of the Nafion 212 membrane (210 μV/h), owing to the weak chemical resistance of the HC membranes to peroxide radicals caused by permeating O_2_ from the anode and cathode. Moreover, HC membranes are vulnerable to interfacial compatibility with the Nafion binder in the CLs, resulting in delamination between the HC membrane and CL when the HC membrane is largely swollen in water during long-term operation [20,41]. For the L-SP50 membrane, the DR was reduced to 290 μV/h compared to that of the SPAES50 membrane because the LCP-nonwoven layer successfully suppressed membrane swelling in the length direction, preventing delamination. These results verify that the SPAES/LCP composite membrane has the potential for use in hydrogen energy devices; however, its DR is still higher than that of the Nafion 212 membrane, confirming that a more chemically stable HC ionomer is needed to enhance the cell efficiency of the HC composite membrane.

## 4. Conclusions

In this study, a new HC composite membrane incorporating LCP-nonwoven fabrics was developed for PEMWE applications. As an HC ionomer, an SPAES ionomer with a sulfonation degree of 50 mol% was synthesized via condensation polymerization. A simple impregnation process was performed to fabricate the composite membrane without any surface treatment of the LCP-nonwoven fabrics. The morphological results showed that the LCP-nonwoven fabrics were well impregnated into the SPAES50 ionomer without noticeable defects. This indicated the successful formation of a physically interlocked structure between the LCP-nonwoven fabrics and SPAES50 ionomers, which improved the mechanical properties and dimensional stability of the membrane. On the other hand, the proton conductivity of the L-SP50 membrane was found to be inferior compared to that of the SPAES50 membrane owing to the non-proton-conductive LCP-nonwoven material. However, in the through-plane direction, the L-SP50 membrane maintained a higher proton conductivity than the Nafion 212 membrane at all the measured temperatures. For the electrochemical properties, the cell performance of the L-SP50 membrane was 5.41 A/cm^2^ at 1.9 V, which was superior compared to that of the SPAES50 membrane (4.65 A/cm^2^), owing to the lower membrane resistance. Moreover, a significantly reduced DR was obtained for the L-SP50 membrane, indicating higher cell efficiency and durability. Consequently, it was demonstrated that the composite membrane consisting of the SPAES50 ionomer and LCP-nonwoven fabrics is a promising candidate for a robust PEM for durable PEMWE applications.

## Data Availability

Not applicable.

## Supplementary Materials

The following supporting information can be downloaded at https://www.mdpi.com/article/10.3390/polym15092109/s1, Figure S1: ^1^H NMR spectrum and degree of sulfonation of the synthesized SPAES50 copolymer; Figure S2: Changes in the length, thickness, and volume of the Nafion 212, SPAES50, and L-SP50 membranes in water; (a) 25 °C, (b) 50 °C, and (c) 80 °C; Figure S3: EIS analysis measuring the resistances (*R_Ω_* + *R_ct_*) of the Nafion 212, SPAES50, and L-SP50 membranes in PEMWE (water flow rate (anode): 30 mL/min; operating temperature: 80 °C; DC potential: 1.7 V; AC frequency: 50 mHz−30 kHz; active area: 5 cm^2^).
