# Supplementary material for: Hydrocarbon-Based Composite Membrane Using LCP-Nonwoven Fabrics for Durable Proton Exchange Membrane Water Electrolysis
Source: Polymers (Basel). 2023 Apr 28;15(9):2109. doi: 10.3390/polym15092109 (PMC10181224; doi:10.3390/polym15092109)
Supplement: Supplementary file 1 [file polymers-15-02109-s001.zip › polymers-2309397-supplementary.pdf]

polymers

MDPI

Supplementary Materials

Hydrocarbon-Based Composite Membrane Using LCP-Nonwoven Fabrics for Durable Proton Exchange Membrane Water Electrolysis

Seok Hyeon Kang [1,†], Hwan Yeop Jeong [1,†], Sang Jun Yoon [1], Soonyong So [1], Jaewon Choi [2,*], Tae-Ho Kim [1,*] and Duk Man Yu [1,*]

[1] Energy Materials Research Center, Korea Research Institute of Chemical Technology (KRICT), Daejeon 34114, Republic of Korea; mlc1207@krict.re.kr (S.H.K.); hwanyeop@krict.re.kr (H.Y.J.); sjyoon@krict.re.kr (S.J.Y.); syso@krict.re.kr (S.S.)

[2] Department of Polymer Science and Engineering, Kyungpook National University, Daegu 41566, Republic of Korea

* Correspondence: jwchoi@knu.ac.kr (J.C.); thkim@krict.re.kr (T.-H.K.); dmyu@krict.re.kr (D.M.Y.)

† These authors contributed equally to this work.

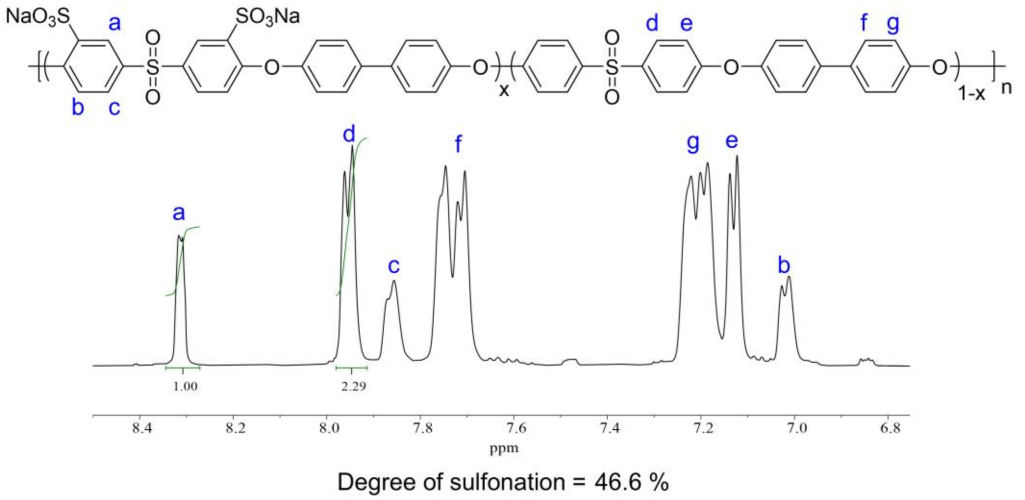

Figure S1. ^{1}H NMR spectrum and degree of sulfonation of the synthesized SPAES50 copolymer.

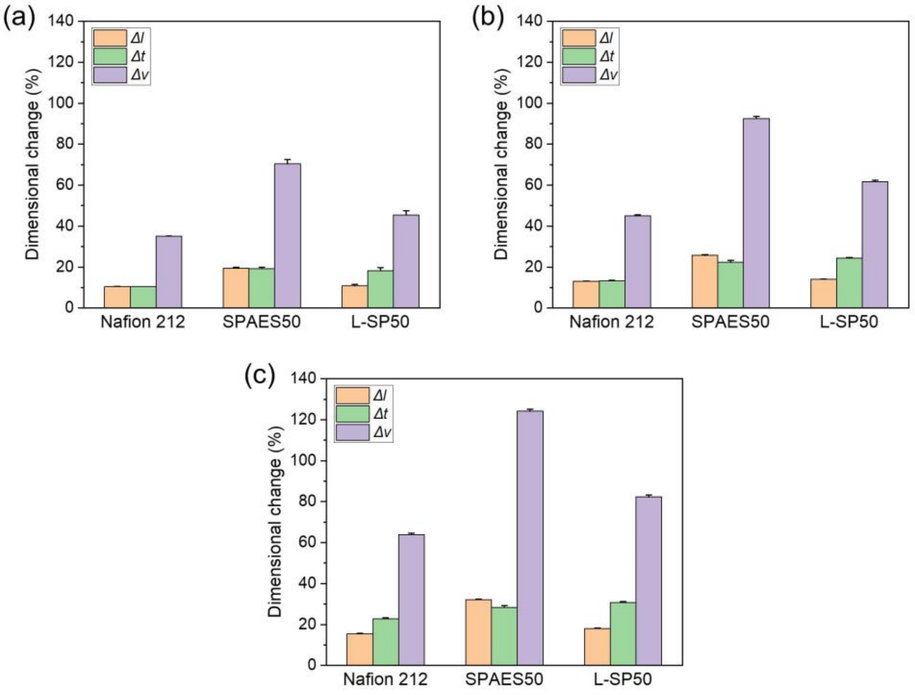

Figure S2. Changes in the length, thickness, and volume of the Nafion 212, SPAES50, and L-SP50 membranes in water; (a) 25 °C, (b) 50 °C, and (c) 80 °C.

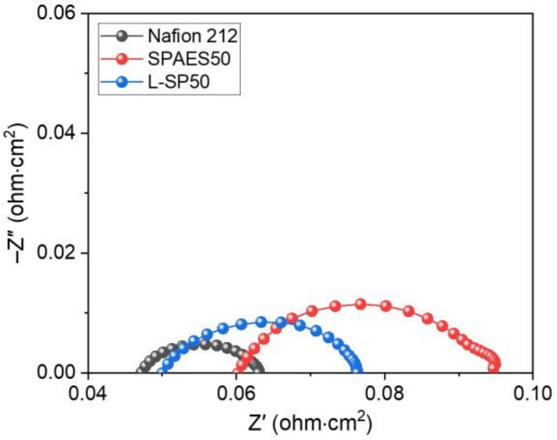

Figure S3. EIS analysis measuring the resistances ($R_{\Omega} + R_{ct}$) of the Nafion 212, SPAES50, and L-SP50 membranes in PEMWE (water flow rate (anode): 30 mL/min; operating temperature: 80 °C; DC potential: 1.7 V; AC frequency: 50 mHz−30 kHz; active area: 5 cm^2).
